# Alkaptonuria with asymmetric otologic involvement: a case report

**DOI:** 10.1016/j.bjorl.2021.03.008

**Published:** 2021-04-08

**Authors:** Ebru Ozer Ozturk, Mehmet Aslan, Mucahit Marsak, Suat Sezer

**Affiliations:** a Inonu University, Faculty of Medicine, Department of Otorhinolaryngology Head and Neck Surgery, Malatya, Turkey; b Inonu University, Faculty of Medicine, Department of Dermotology, Malatya, Turkey

## Introduction

Alkaptonuria is a rare autosomal recessive disease, which is caused by mutations in the homogentisate 1.2-dioxygenase gene on chromosome 3q13.^1^ This metabolic disorder results from the deficiency of homogentisic acid (HGA) oxidase that is responsible for the breakdown of HGA, an intermediate product in the tyrosine degradation pathway.^2^ The consequent increase in levels of HGA causes polymerization and accumulation of a melanin-like pigment that is selectively deposited in connective tissues.^3^ This pigment has a high affinity for skin, bone, and mucosal surfaces, hyaline cartilages, intervertebral discs, skin, sclera, and the concha and helix of the ear.^4^, ^5^ Otologic involvement has previously been described by one study and limited number case reports.^5^, ^6^

We report systemic, otoscopic and audiological findings for a patient with alkaptonuria. Interestingly, otoscopic examination revealed symmetrical auricular involvement and unilateral tympanic membrane involvement.

## Case report

A 62-year-old female patient admitted to the dermatology clinic for discoloration of the auricle for 2 years. The patient was consulted by otorhinolaryngology for discoloration in the auricle. The patient had no history of otological surgery, infection, trauma, otorrhea, or tympanic membrane perforation. Upon questioning, the patient had morning stiffness and low back pain for many years. She was diagnosed with ankylosing spondylitis in 2012.

**Figure 1:**
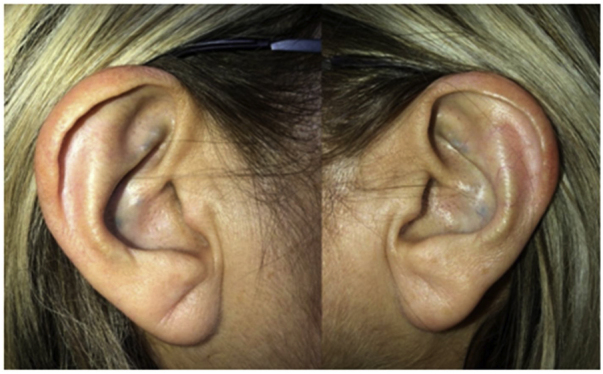
Bilateral green-black pigmentation of auricula.

**Figure 2:**
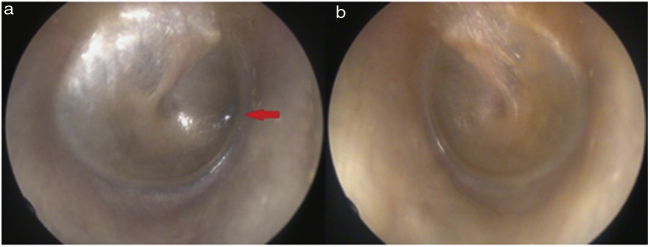
Unilateral tympa nic membrane involvement, green-black pigmentation in the right tympanic membrane.

On physical examination, green-black pigmentation was seen in the crura antihelix, and concha auricularis in the bilateral auricle (Fig. 1). Otoscopic examination revealed green-black pigmentation on the right tympanic membrane, while the left tympanic membrane was in normal appearance (Fig. 2). High frequency hearing loss was detected in the audiometric examination. Tympanometric examination revealed type A tympanograms bilaterally, and the absence of acoustic reflexes. Computed tomography of the temporal bone showed no pathology.

**Figure 3:**
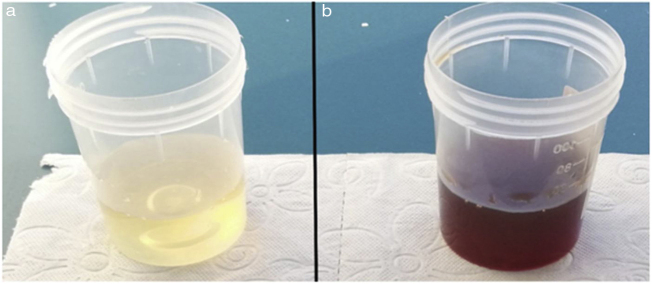
The normal colored urine of the patient turned in to black after alkalinization with KOH.

The urine had a typical dark appearance after alkalinization with KOH (Fig. 3). Relevant laboratory results included a urine organic acid screen with considerably elevated HGA excretion (670 mmoL/moL creatinine). The genetic screening revealed combined heterozygous c.175deIA/p.Ser59AIafsTer52 and c.674 G > A/p.Arg225His mutations in the exons of homogentisate 1.2-dioxygenase gene. Renal and liver function tests, electrocardiogram and echocardiogram were within normal limits. As a result of the relevant examinations and tests, the patient was diagnosed with alkaptonuria.

## Discussion

Alkaptonuria has three distinct features which are homogentisic aciduria, ochronotic osteoarthropathy, and ochronosis.^3^ Each feature presents at a different stage in life. Homogentisic aciduria is present at birth and can lead to early diagnosis by the darkening of the urine in infancy.^3^ Ochronosis, which occurs secondary to polymerization of HGA, appears between the third and fifth decade of life with melanin-like pigment deposition in the ears and eyes.^1^ Ochronotic osteoarthropathy arises about the fourth decade and mostly affects the large weight-bearing joints, such as the knees, leading to changes similar to osteoarthritis radiographically.^7^ The vertebrae are also often affected, with osteophyte formation and stiffening of the intervertebral joints.^3^ Furthermore, affected patients are at an increased risk of bone fractures, nephrolithiasis, prostate stones, and valvular heart disease.^8^

The majority of patients are asymptomatic until the third or fourth decade of life and causes symptoms with the onset of ochronosis.^8^ Steven et al. evaluated 20-patients with alkaptonuria, and they reported that the average age at diagnosis was 24 (range 0–65) years.^6^ In our case, since the symptoms occurred late, the alkaptonuria diagnosed in the sixth decade. This patient admitted to the dermatology clinic for green-black discoloration in the ear without a diagnosis of alkaptonuria, the diagnosis was made after the relevant examinations. Our patient had morning stiffness and low back pain for several years and was diagnosed with ankylosing spondylitis eight years ago. These symptoms may have resulted from secondary to ochronotic osteoarthropathy.

Steven et al. reported ENT findings in alkaptonuria patients.^6^ They observed ENT signs or symptoms in eighteen of the 20 patients (90 percent). Otological signs or symptoms observed in this study: pinna discoloration 60%, cerumen discoloration 65%, tympanic membrane discoloration 10%, middle ear discoloration 5%, otalgia 15%, tinnitus 30% and high frequency hearing loss 50%.^6^ Our report reveals green-black pigmentation on the right tympanic membrane and helixes of both ears, in addition to previously diagnosed lower back involvement. Our case also had mixed high frequency hearing loss. The conductive part of this hearing loss might have developed as a result of progressive ochronotic pigment deposition and resulting altered tympanic membrane elasticity or as a result of ochronotic impairment of the ossicular joint mobility.

Because of renal excretion of HGA, high urinary HGA levels are diagnostic of alkaptonuria.^8^ Furthermore, detection of the defect occurred in the gene which codes homogentisate 1.2 dioxygenase located on the 3q chromosome support the diagnosis of alkaptonuria. Pigmentation of the ear auricle, tympanic membrane and sclera, and/or altered urine color, arthropathies are typical clinical manifestations and can aid diagnosis.

Different therapeutic modalities have been applied in the treatment of alkaptonuria, such as ascorbic acid, nitisinone, and a low protein diet.^9^ The nitisinone has the potential to modify the disease by reducing homogentisic aciduria.^6^ It is, therefore, essential to detect alkaptonuria early so that patients may be offered treatment.

## Conclusion

Despite being rare, alkaptonuria patients can present to the otolaryngologist. The otolaryngologist should be aware of the ENT findings of alkaptonuria because successful early diagnosis and referral to a relevant specialist are crucial so that disease-modifying treatment such as nitisinone can be administered.

## Conflicts of interest

The authors declare no conflicts of interest.
